# Acute Progression of Adult-Onset Atypical Hemolytic-Uremic Syndrome due to CFH Mutation: A Case Report

**DOI:** 10.1155/2013/739820

**Published:** 2013-02-21

**Authors:** Bartlomiej Posnik, Dorota Sikorska, Krzysztof Hoppe, Krzysztof Schwermer, Krzysztof Pawlaczyk, Andrzej Oko

**Affiliations:** Department of Nephrology, Transplantology and Internal Medicine, Poznan University of Medical Sciences, 60-355 Poznan, Poland

## Abstract

Atypical hemolytic-uremic syndrome (aHUS), unlike typical HUS, is not due to bacteria but rather to an idiopathic or genetic cause that promotes dysregulation of the alternative complement pathway. It leads to hemolytic anemia, thrombocytopenia, and renal impairment. Although aHUS secondary to a genetic mutation is relatively rare, when occurring due to a mutation in Factor H (CFH), it usually presents with younger onset and has a more severe course, which in the majority ends with end-stage renal failure. Paradoxically to most available data, our case features acute aHUS due to a CFH mutation with late onset (38-year-old) and rapid progression to end-stage renal disease. Due to current data indicating a high risk of graft failure in such patients, the diagnosis of aHUS secondary to a genetic cause has disqualified our patient from a living (family) donor renal transplantation and left her with no other option but to begin permanent renal replacement therapy.

## 1. Introduction

Hemolytic-uremic syndrome (HUS) is characterized by hemolytic anemia, thrombocytopenia, and renal failure. It is most frequently caused by Shiga-like toxin bacterial infections in the digestive tract, such as from *Escherichia coli* [1]. Atypical HUS (aHUS) refers to non-Shiga-toxin HUS, a primary disease due to a disorder of alternative complement pathway regulation; it represents only 5–10% of HUS in children, but the majority in adults [2]. aHUS is a rare renal disease, about two per one million in the United States, with 80% of cases due to a sporadic and 20% a familial form [2, 3]. 

Diagnosis of aHUS requires the exclusion of other associated diseases, a lack of criteria for typical HUS, and a lack of criteria for thrombotic thrombocytopenic purpura determined with serum ADAMTS 13 activity [2]. Historically, these patients tend to have a poorer prognosis than those with typical HUS, with an acute aHUS mortality of 8% [4], and with 50%–80% of patients progressing to end-stage renal failure. Individuals with aHUS frequently relapse even after complete recovery from the presenting episode; this course of illness is more likely to be genetic in origin [1]. In patients with aHUS, mutations were reported in the genes of three proteins that regulate the alternative complement pathway: Factor H (CFH), membrane cofactor protein (MCP or CD46), and Factor I (IF) [5–8]. Plasma infusion or exchange has been done to reduce mortality, and patient surveillance should be continued on a regular basis to check for markers of renal failure and problems with RBC, hemoglobin (Hb), and platelet concentrations [1]. 

Since most current research pertains to childhood onset and its predisposing genetic factors, there seems to be a general lack in research regarding the onset, course, and severity of this disease among the adult population. Therefore, little is known about the typical course of genetically verified aHUS in adulthood, as presented in this paper.

## 2. Case Presentation

A 38-year-old female presented to her GP with symptoms typical of an upper respiratory tract infection. Clindamycin treatment was initiated without any signs of improvement. Patient history demonstrated only hypertension of two years duration and a family history of cardiovascular disease. With a suspicion of endocarditis, and with worsening symptoms of dyspnea, fatigue, decreased physical tolerance, and tachycardia, the patient was hospitalized. Taking into consideration the patient's physical symptoms, poor kidney function (creatinine > 5 mg/dL and urea > 200 mg/dL), severe anemia (Hb 7.0 g/dL), thrombocytopenia (60 × 10^3^/*μ*L), and fragmented red blood cells on peripheral smear, a diagnosis of hemolytic-uremic sdyndrome (HUS) was established. Due to a continued drop in renal function (creatinine 7.0 mg/dL, urea 230 mg/dL), a short-term Shaldon catheter was placed and hemodialysis (standard 3 hour) with concurrent plasmapheresis and plasma transfusion was started and repeated until a volume of 4 liters of donor plasma was used. The patient was also given therapy with three pulses of methylprednisolone, which yielded no improvement.

Upon arrival at the nephrology department in Poznan, the patient's condition was considered severe with dyspnea at rest, tachycardia, hypertension (190/100 mmHg), oliguria, decreased renal function (creatinine 4.69 mg/dL, urea 124 mg/dL), hemolysis (LDH 982 U/L), and severe anemia and thrombocytopenia (Hb 7.9 g/dL, RBC 2.56 × 10^6^/*μ*L, platelets 60 × 10^3^/*μ*L). Ultrasound examination revealed bilaterally enlarged kidneys with swollen cortices of slightly increased echogenicity, and chest X-ray revealed fluid in the left pleural cavity. Treatment was continued with the standard 3-hour hemodialysis and plasmaphoresis (using 4 liters of donor plasma), as well as intensive pharmacological treatment for hypertension with urapidil, metoprolol, clonidine, amlodipine, doxazosin, furosemide, and losartan. After two courses of hemodialysis, diuresis was reestablished and a halt in worsening of renal failure indicators was observed. Plasmaphoresis was done a total of 15 times during hospitalization, and a single three-unit RBC transfusion was also administered because of continued anemia (Hb 6.5 g/dL). After 24 days of treatment, the patient's condition was stabilized yielding normal diuresis and blood pressure as well as an improvement in renal function and blood values, and the patient was released home. 

Disease remission occurred 3 months after initial presentation with worsening hypertension despite treatment and a 3-week history of lower limb edema. The patient was noted to be in stable condition but with tachycardia, hypertension (150/90 mmHg), increased creatinine (3 mg/dL), increased urea (109 mg/dL), bilateral lower limb edema, decreased blood values (Hb 7.4 g/dL, RBC 2.68 × 10^6^/*μ*L, platelets 107 × 10^3^/*μ*L), and severe hemolysis (LDH 377 U/L). Treatment was once again with hemodialysis and plasmaphoresis, and at this time a decision was made to determine the presence or absence of an ADAMTS 13 mutation. Samples were taken 2 weeks after plasmaphoresis and yielded negative results (Table 1).

Over the next 7 months, the patient was hospitalized an additional 7 times during which she underwent only plasmaphoresis 2-3 times per recurrence, again with a total of 4 liters of plasma, as well as an ancillary tonsillectomy to rule out the tonsils as a possible source of infection. Due to a continued decrease in renal function, based on increased levels of creatinine and urea, evaluation began to determine qualification for renal transplantation. Among the tests performed was an additional genetic exam, which demonstrated a heterozygous mutation of Factor H (Table 2). Due to this result, the patient was disqualified from a living (family) donor renal transplantation and was entered into a permanent hemodialysis program. At the time of this paper, the patient is in preparation for treatment with peritoneal dialysis.

## 3. Discussion

After all clinical and laboratory evaluations, the diagnosis of aHUS was concluded in our patient due to the exclusion of other associated diseases, exclusion of typical HUS, and exclusion of ADAMTS 13 mutation as per criteria defined by Loirat and Frémeaux-Bacchi [2]. Atypical HUS is a rare renal disease, about two per one million in the United States, with 80% of cases due to a sporadic form and 20% due to a familial form [2, 3]. Onset ranges from neonatal age to adulthood [1], but most studies focus on early onset of the disease. One of the most cited studies on this topic, by Constantinescu et al. [9], is based on a mean age of onset of 4.2 years. This study reported that aHUS incidence is one-tenth that of typical HUS and that these patients tend to require more hemodialysis treatments and longer hospital stays. These observations are consistent with our patient, as she had to undergo frequent hemodialysis due to overhydration and her rising levels of creatinine and urea. Nonetheless, a lack of concrete research exists regarding a later age of onset and its effect on duration and/or severity of the disease. Historically, these patients tend to have a poorer prognosis than those with typical HUS [4]. However, at least one report describes cases of aHUS having a prognosis similar to that of typical HUS [10], although this data once again is derived from children and not adults.

CFH mutations, usually missense, account for 30% of aHUS [1], and patients with such mutations were found to have the earliest onset and the highest mortality [11], an observation that does not correspond with our patient's age at onset. There are more than 70 mutations in CFH linked to HUS patients (http://www.fh-hus.org/) and the one found in our patient located at SCR20 (Exon23) due to a single nucleotide polymorphism C3572G missense (Table 2) has been described in patients affected by aHUS [12]. This determination of a genetic cause of aHUS is important prior to renal transplantation since recurrence is observed in 60% of patients of which 91.6% went on to develop graft failure [1, 13]. The presence of a CFH mutation in particular was found as being associated with an even higher incidence of graft failure at 77.8% versus 54.9% in patients without the CFH mutation [13]. The incidence of graft failure was also found to be higher in adults (62.5%) then in children (42.8%) [13]. This suggests that transplantation is not necessarily a cure but rather a treatment of undetermined duration and is thus evidence towards justification of our patient's disqualification from receiving a renal transplantation from a living (family) donor. This is a heavily debated topic especially in light of other studies as the one performed by Dragon-Durey et al. [14] in which renal transplant in patients with homozygous or heterozygous CFH deficiency was performed yielding success in 3 and failure in only 1 due to recurrence. However, the majority of studies performed on this topic tend to demonstrate a poor kidney graft prognosis with more than 50% lost due to recurrence [13, 15–17]. Once again, a shortcoming of the available data, especially with successful outcomes, is that it pertains mainly to children and not adults.

## 4. Treatment

Currently the mainstay of aHUS treatment relies on plasmaphoresis, and it is stated that mortality rate has decreased by half since the introduction of plasma manipulation and that a reliable amount of HUS patients respond to this therapy [18]. In cases similar to our patient's with renal insufficiency, it has been found that plasma exchange is beneficial over plasma infusion alone [19]. According to Noris et al. [1], plasma count and serum LDH concentration are the most sensitive markers in determining the success of plasma therapy; therefore, treatment was beneficial in our patient already during her first hospitalization in our department during which time her platelet and LDH levels improved from 60 × 10^3^/*μ*L and 982 U/L at admission to 219 × 10^3^/*μ*L and 169 U/L at discharge, respectively. Success of hemodialysis was determined by the disappearance of symptoms such as lower limb swelling and dyspnea, as well as a decrease in creatinine and urea levels. Consistently with Noris et al. [11], our patient was within the 60% that underwent partial or complete remission after plasma exchange. Unfortunately the remission was brief, and it was eventually decided that due to a lack of prolonged remission, increasing levels of serum creatinine and urea, and a disqualification from a living (family) donor renal transplantation, our patient was added to the permanent hemodialysis program. Currently, measures are being taken to begin renal replacement therapy using peritoneal dialysis since it boasts clear advantages in patients of working age [20, 21]. Although not used in this case because of a lack in availability, new studies show promise in treating aHUS with the use of Eculizumab, an anti-C5 monoclonal antibody, which prevents activation of the terminal complement cascade [22]. Recent data suggests that while Eculizumab shows immediate improvements, it may require long-term use since dose reductions and discontinuation have demonstrated rapid deterioration in organ function [23].

## Figures and Tables

**Table 1:** Results of ADAMTS 13 testing.

	Patient's results	Normal reference range
ADAMTS 13 activity (%)	68.77	50–150
ADAMTS 13 antigen (ug/mL)	0.94	0.60–1.60
ADAMTS 13 inhibitors (U/mL)	5.31	<12

**Table 2:** Genetic analysis of Factor H (CFH).

CFH	SCR15	SCR16	SCR17	SCR18	SCR19	SCR20
Patient	CC	GG	Norm	CC	Norm	CG
SNP	C2634T	G2808T		IVS21 + 144C > T		C3572G
Amino acid	His878His	Glu936Asp				Ser1191Trp

SCR: short consensus repeat; SNP: single nucleotide polymorphism. Results obtained from Istituto Di Ricerche Farmacologiche Mario Negri, Ranica, Italy.
